# Morphology on Reaction Mechanism Dependency for Twin Polymerization

**DOI:** 10.3390/polym11050878

**Published:** 2019-05-14

**Authors:** Janett Prehl, Constantin Huster

**Affiliations:** Institut für Physik, Technische Universität Chemnitz, D-09107 Chemnitz, Germany; constantinhuster@web.de

**Keywords:** twin polymerization, radial distribution function, specific surface area, porosity, percolation, Monte Carlo method, reactive bond fluctuation model

## Abstract

An in-depth knowledge of the structure formation process and the resulting dependency of the morphology on the reaction mechanism is a key requirement in order to design application-oriented materials. For twin polymerization, the basic idea of the reaction process is established, and important structural properties of the final nanoporous hybrid materials are known. However, the effects of changing the reaction mechanism parameters on the final morphology is still an open issue. In this work, the dependence of the morphology on the reaction mechanism is investigated based on a previously introduced lattice-based Monte Carlo method, the reactive bond fluctuation model. We analyze the effects of the model parameters, such as movability, attraction, or reaction probabilities on structural properties, like the specific surface area, the radial distribution function, the local porosity distribution, or the total fraction of percolating elements. From these examinations, we can identify key factors to adapt structural properties to fulfill desired requirements for possible applications. Hereby, we point out which implications theses parameter changes have on the underlying chemical structure.

## 1. Introduction

In the last years, the design and development of simple process pathways for application-oriented materials has came up as an important field of research [1,2,3,4,5,6] in material science, chemistry, polymer engineering, and polymer technology. Especially in the topic of adapting materials to specific requirements of applications, the analysis of the morphology and an in-depth knowledge of the occurring structure formation processes induced by the reaction mechanism or the manufacturing process are key features to understand and afterwards to design materials for desired requirements.

**Figure 1:**
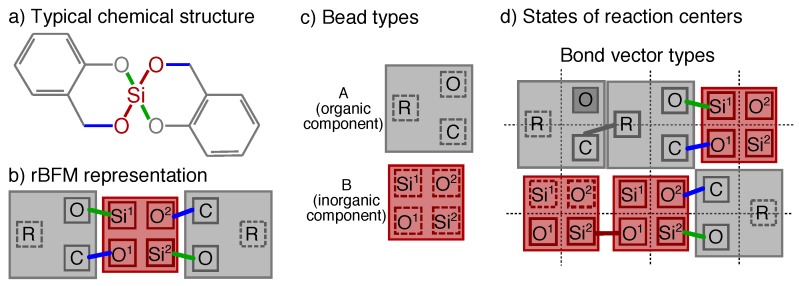
The chemical structure of the typical twin monomer 2,2${}^{\prime }$-spiro[4*H*-1,3,2-benzodioxasiline] is shown in (**a**), and its reactive bond fluctuation (rBFM) representation in 2D in (**b**). It is represented by two bead types (A,B) and five different reaction centers (O,C,R,O*^x^*,Si*^x^*, with x={1,2}) as illustrated in (**c**,**d**). In (**d**) all possible states of the reaction centers and all possible bond vector types are given in an exemplary 2D-cutout of the cubic lattice. A corresponding 3D representation is given in Figure 2. Reaction centers with a solid square are bonded, with a dashed square are non-bonded and with darker filling are blocked. Note that to make it easier to differentiate between the reaction centers that are all placed in the center of the beads, here they are plotted next to each other.

One recently developed simple synthesis route to create nanostructured hybrid materials is twin polymerization [6,7]. Twin polymerization starts with twin monomers consisting of at least two different components, an organic and an inorganic one, that are connected via two differently behaving bonds. An exemplary twin monomer is shown in Figure 1a, where the different components and bonds are highlighted in different colors. Due to the complex reaction mechanism these twin monomers react acid-, base-, or thermally induced into two separated but highly interweaved organic and inorganic networks with typical domain sizes of 0.5 to 3 nm [8,9]. By simple post-processing [10], a microporous organic phenolic resin or mesoporous inorganic silica network can be obtained for various applications. It appeared that these materials are of special interest in the fields of anodes for accumulators and gas adsorption systems [11] or for lithium ion batteries [12].

To modify the obtained twin polymer structure, a wide class of different twin monomers has been introduced and various approaches and several experimental studies have been conducted [6,7,13]. However, a detailed understanding of the morphology to reaction mechanism dependency is still missing. Important questions like: “Which elements of the twin monomer structure need to be changed to obtain an nanoporous hybrid materials with a desired pore size distribution?” or “Where to tune the reaction mechanism to generate a specific application based material with desired specific surface area?” are still difficult to answer. To bridge this gap, in the last years several theoretical approaches [9,14,15,16,17,18,19] at different levels of detail have been established. From these investigations, we gain important insights into the reaction mechanism of the thermal, acid- and base-induced twin polymerization. Furthermore, a reactive bond fluctuation model (rBFM) [9,18,19] has been derived which connects the structure formation process of the reaction mechanism with the macroscopic structural properties of the synthesized twin polymers. It showed that important structural properties of the twin polymers, i.e., the pore size distribution [9], are reproduce in very good agreement with experiments.

The rBFM [9,18,19] is an extended lattice-based bond fluctuation model [20,21,22]. It opens up the possibility to systematically investigate the effects of all reaction relevant parameters and to evaluate their impact on the final morphology. With this knowledge we can deduce a) the parameters that need to be adapted to achieve a specific change in the final structure and b) their implications on the underlying chemical structure. The main focus in this work will be the extraction of major influence factors on structural properties such as bond formation, phase separation, bulk and local porosity, specific surface area, radial distribution function and percolation of the final material. This will be done for the organic–inorganic hybrid materials, as well as for the organic and the inorganic networks theirselves. With this knowledge one will be able to derive chemical characteristics that needs to be fulfilled to synthesize specific morphologies, or specific applications.

The paper is structured as follows: First, we propose the rBFM and its components, second, the twin polymerization and its mapping to the rBFM, and third, all reaction relevant and structural properties that will be investigated. We will introduce and explain all examined model parameters. Then, we will present the obtained results and discuss them. During the discussion, we will identify the major influence factors to modify the final morphology. At the end, a summary of the overall results and possible perspectives for further research are given.

## 2. Numerical Model, Materials, and Methods

### 2.1. Reactive Bond Fluctuation Model

The reactive bond fluctuation model (rBFM) is a Monte Carlo method that is based on the bond fluctuation model (BFM) known from literature [20,21,22]. Within in BFM chemical structures, like polymers and macromolecules, are represented not by their atoms and interacting forces, but by coarse grained elements that can move with certain probabilities on a (typically cubic) lattice. In our extension, more complex connections between coarse grained elements as well as reactions, i.e., bond formation and cleavage, are included additionally.

**Figure 2:**
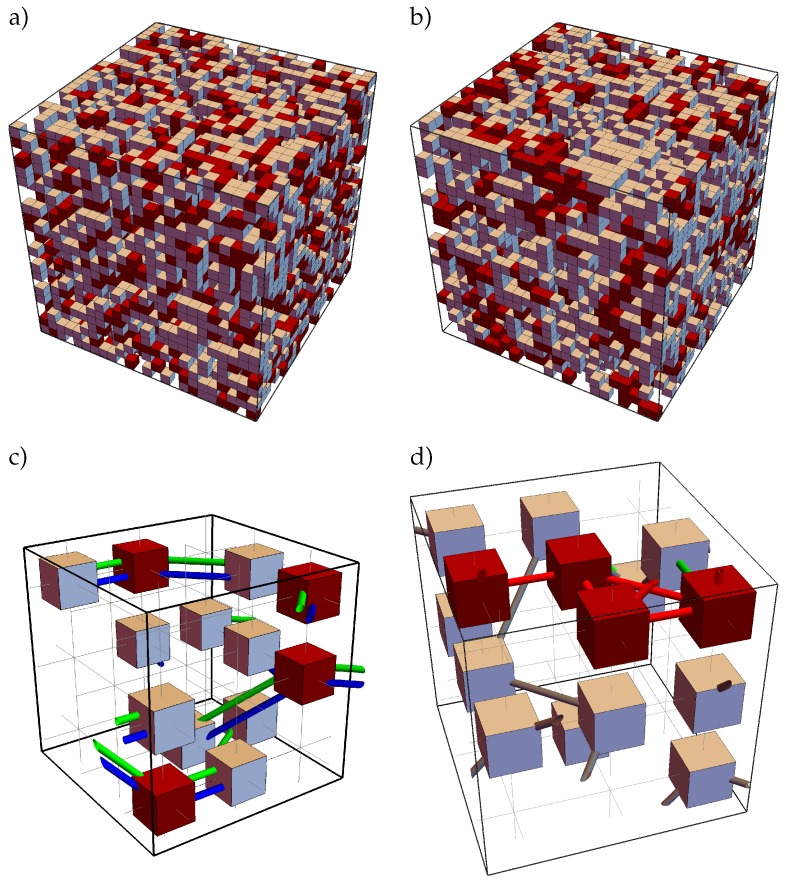
Exemplary snapshots of a reduced 3D cubical lattice of reduced size 24×24×24 at (**a**) $t_{\mathrm{ini}}$ and (**b**) $t_{\mathrm{fin}}$ for the parameter combination (I) (see Equation (9)) and two corresponding cutouts of size 6×6×6 (**c**): $t_{\mathrm{ini}}$; (**d**): $t_{\mathrm{fin}}$) that are magnified to show the actual existing bond vectors. The colors are chosen in analogy to Figure 1. In (**c**) only twin monomer structures are observed (see Figure 1b). As over reaction time bonds may cleave and form, a final structure emerge as depict in (**d**), where a small organic (gray gray) and inorganic (red bonds) network emerge. Note that some initial bonds (green) may survive till the end. The origin of both sub cubes is at position {6,6,6} within the large 3D cubes. Note that to make it easier to differentiate between the bond vector types that are connected to the center of the beads, here they are plotted next to each other.

Chemical structures that do not alter there steric structure during the reaction process are merged to cubic beads in the framework of the rBFM. Each bead can contain several reaction centers in the center of the beads, which are the connection points between two beads and which are accessible from all sides, i.e., orientation is nonrelevant. A reaction center represents a typical chemical element of the original chemical structure where a bond may form or cleave. A reaction center can take different states like bonded, non-bonded or blocked. The bonds are represented by bond vector types, which are the shortest distance vector between two reaction centers. A bond vector type is defined via the two reaction centers it connects and each bond vector type has an allowed set of different finite lengths, depending on the underlying chemical structure [21,23,24]. In Figure 1 an exemplary structure of a typical twin monomer 2,2${}^{\prime }$-spiro[4*H*-1,3,2-benzodioxasiline] (**1**) and its mapping to possible bead types, reaction centers, and bond vector types is shown. The corresponding 3D representation of such a structure before and after an exemplary reaction is shown in Figure 2. Note that in Figure 1 and Figure 2 for a better differentiation the reaction centers, and bond vectors are plotted next to each other instead of in the center of the beads.

The beads can move on the sites of cubic lattice L of size L×L×L, with an edge length *a* (lattice constant) between two neighboring sites. Here, we use the lattice notation of Shaffer [21], i.e., the beads move on the even grid lattice nodes, whereas on the odd grid lattice nodes the bond crossings are counted. For structure determination only the even grid lattice nodes are taken into account, which will be called reduced lattice. Typically, periodic boundary conditions are defined, but others are also possible. Each bead occupies one lattice site. Double occupation of lattice sites as well as crossing of bonds is prohibited. The beads can perform non-reactive Monte Carlo (nMC) steps. This means a bead can move to a neighboring site if

- the chosen lattice site is empty,
- all final bond lengths are allowed, and
- the final bonds do not cross each other.

In order to include possible effects like phase separation, as it occurs in immiscible polymer blends [25,26] or systems with different hydrophobicities [27], we introduce an attraction parameter λ to allow different pairwise interaction energies between identical (αα) and different (αβ) bead types. Therefore we define a rate constant for the movability as $k_{\mathrm{move}}=k_{0}\;e^{\beta \;\Delta E}$ with $k_{0}$ as prefactor, β is the inverse temperature $1/(k_{B}T)$ and ΔE as energy difference between the system before and after the potential move, to move a bead one allowed position on the lattice. Transferring this approach to the rBFM we obtain

(1) $$\begin{array}{cc} k_{\mathrm{move}} & =k_{0}\;e^{\lambda \;[\left(n_{\alpha \alpha }\left(t\right)-n_{\alpha \beta }\left(t\right)\right)-\left(n_{\alpha \alpha }\left(t-1\right)-n_{\alpha \beta }\left(t-1\right)\right)]}, \end{array}$$

with the attraction parameter λ∈R representing β and the energy difference ΔE in units of $k_{B}T$ given by the number of neighboring beads of identical or different bead type $n_{\alpha \alpha }$ and $n_{\alpha \beta }$ before (t−1) and after (t) the nMC step. From this we can derive the metropolis probability to accept the move as

(2) $$\begin{array}{cc} p_{\mathrm{move}} & =\min\left\{1,\frac{k_{\mathrm{move}}}{k_{0}}\right\}=\min\left\{1,e^{\lambda \;[\left(n_{\alpha \alpha }\left(t\right)-n_{\alpha \beta }\left(t\right)\right)-\left(n_{\alpha \alpha }\left(t-1\right)-n_{\alpha \beta }\left(t-1\right)\right)]}\right\}\;. \end{array}$$

Note that in the case of λ=0, all neighboring lattice sites are chosen randomly from a uniform distribution with equal probability for the nMC step. Furthermore, we do not differentiate for λ between αα, αβ, βα, and ββ in order to keep the model as simple as possible but as complex as necessary. Our choice means that beads of the same type attract each other equally strong for both types and that beads of different bead types shows a repulsion strength of the same size. The interaction energy can be attractive or repulsive depending on the local environment as indicated by Equation (2). The simplest version of attraction and repulsion is also studied in [28].

Parallel to the nMC steps the beads can also perform reactive Monte Carlo (rMC) steps. Therefore, first, an initial bead, second, a possible reaction for this bead, and third, a reaction partner (second bead) within its surrounding is chosen randomly. If

- the reaction is allowed and
- no crossing of bonds occur after the rMC step,

the rMC steps is accepted with a metropolis rate $p_{\mathrm{reac}}$, which will be called reaction probability in the following.The ratio of non-reactive to reactive MC steps can be varied via the ratio *m*.

### 2.2. Twin Polymerization

We start our systematical investigation of the structure formation process by briefly reviewing the reaction mechanism of twin polymerization for 2,2${}^{\prime }$-spiro[4*H*-1,3,2-benzodioxasiline] and its mapping to the rBFM.

**Figure 3:**
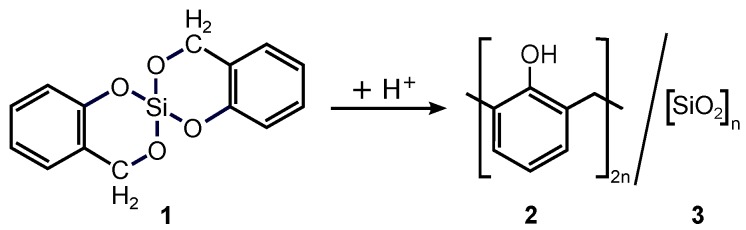
The acid catalyzed reaction scheme of the typical twin monomer 2,2${}^{\prime }$-spiro[4*H*-1,3,2-benzodioxasiline] (**1**) is shown. **1**twin polymerizes to a phenolic resin (**2**) and a silica network (**3**).

The rBFM is intentionally established to investigate the acid catalyzed twin polymerization of the typical twin monomer 2,2${}^{\prime }$-spiro[4*H*-1,3,2-benzodioxasiline]. As shown in Figure 3 **1** twin polymerizes to a phenolic resin (**2**) and silica network (**3**). From experimental [6,7,8,10,29,30] and theoretical [8,14,15,16,17,18] investigations it is known, that the acid catalyzed reaction mechanism is a reaction in a melt [8]. It can be characterized by three main reaction steps. First, the methylene bonds open (ring opening process), second, the organic network formation via organic bond formations starts, and third, somehow later the inorganic network formation starts by opening the aryl bonds and forming the siloxane bonds at the same time. For further details to the reaction mechanism we refer the reader to [17,18,19].

Mapping the twin polymerization of **1** to the rBFM (see Figure 3), leads to two different types of beads A and B that can be identified with the organic and inorganic component of **1**, respectivly. The organic bead type A consists of three reaction centers, C, O, R, whereas the inorganic bead type B has two times two different reaction centers, Si*^x^*, O*^x^*, as indicated by the index x={1,2}. Note that all reaction centers despite O have two states, bonded and non-bonded, whereas O can also be blocked, as depict in Figure 1.

Due to the reaction mechanism of **1**, we introduce four different bond vector types, i.e., methlyene bond (OSi*^x^*), aryl bond (CO*^x^*), organic bond (CR), and siloxane bond (Si*^x^*O*^x^*), which can take the bond vector lengths $\left\{1,\sqrt{2},\sqrt{3}\right\}$. We allow the following reactions during the rMC step:

- cleavage of OSi*^x^*,
- formation of CR,
- cleavage and formation of CO*^x^*, and
- formation of Si*^x^*O*^x^*.

For our further analysis we keep the basic structure of the original twin monomer **1**, as it includes all important features of a twin monomer, i.e., an inorganic and organic components that are connected via two differently behaving bonds, as well as the reaction mechanism itself. However, we varied all reaction probabilities, the ratio *m* of non-reactive to reactive MC steps, and the attraction parameter λ. Within Table 1 the varied model parameters, their symbols and the range of variation are specified.

Hereby it is important to note that the variation of the model parameters can be identified with the following changes in the structure of the twin monomer or the real experimental setup. Modifying the reaction probabilities means a change of the bonding energies, which could be achieved by changing the underlying chemical structure, i.e., replacing atoms. Adjusting *m* is used as a basis of discussion how diffusive effects influence the structure formation process. The parameter λ does also represent a change of the chemical structure of beads, so that the single components are more or less attractive among each other.

### 2.3. Simulation Details

For a typical simulation run, we place *N* beads, i.e., $N_{A}=2\;N/3$ and $N_{B}=N/3$ beads, randomly on the cubic lattice, where always three beads are arranged as shown in Figure 1b. We choose *N* as $N=L^{3}\;\tilde{\Phi }$ with the volume fraction $\tilde{\Phi }=0.5$. This volume fraction represents a dense melt in the bond fluctuation model [20]. An exemplary filled cubic lattice of reduced size 24×24×24 is given in Figure 2a and a corresponding cutout of size 6×6×6 in Figure 2c.We checked the influence of the system size by performing the simulations for L={48,72} with a=1 (lattice notation as Shaffer [21]). We observed no qualitative and quantitative changes of the quantities under investigation.

The reaction time is measured in Monte Carlo Cycles (MCCs). In one MCC each bead can perform randomly a reactive or non-reactive MC step. The ratio *m* can be varied but it is fixed per simulation run. For the first 1000 time-steps the rMC steps are switched off, thus the system can relax. Afterwards, the rMC steps are switched on and the reaction process starts. Note that we always use the time step before the rMC steps are switched on as initial time $t_{\mathrm{ini}}$. The simulation ends when the relative changes of number of reaction centers in each state are not larger than 5 × 10${}^{-5}$ over 10 successive time steps on the logarithmic time scale. The final time of each simulation is $t_{\mathrm{fin}}$. An exemplary final cubic lattice of reduced size 24×24×24 is given in Figure 2b and a corresponding cutout of size 6×6×6 in Figure 2d.

During the simulation the number of reaction centers in each state and the positions of all beads are tracked over time. This allows us to count the number of the non-bonded reactions centers C–, O–, R–, O*^x^*–, Si*^x^*– and of the bond vectors CO*^x^*, OSi*^x^*, CR, Si*^x^*O*^x^*. In following these reaction centers and bonds are indicated by ξ. Additionally we also determine the number of all nearest-neighbors (${\hat{n}}_{\alpha \alpha }\left(t\right)$, ${\hat{n}}_{\alpha \beta }\left(t\right)$) and next-nearest-neighbors ($n_{\alpha \alpha }\left(t\right)$, $n_{\alpha \beta }\left(t\right)$) per time step, where αα represents counts between identical bead types and αβ between different bead types.

### 2.4. Process to Structure Analysis

In previous work [9,18,19] we showed that with a proper choice of parameters the rBFM is capable to reproduce the typical three-phase-reaction behavior of the twin polymerization of **1** [18,19], as one observes the initial ring opening phase, the organic, and the inorganic network formation phase, as supposed by previous findings [8,14]. And it can reproduce qualitatively the correct morphology of the final nanoporous hybrid material, as for instance shown for the pore size distribution [9].

In order to qualitatively understand the effects of the model parameters of the rBFM on the final morphology we will perform a systematic analysis of all effects and a discussion of the corresponding consequences in the following. Interesting questions that we want to answer are: Which parameter lead to which changes in the morphology? Is there a different effect on the full nanoporous hybrid material, than on the organic or inorganic components itself? Does the same parameter combination lead to different effects for the different structural properties? Therefore we define chemical properties that quantify the outcome of the reaction mechanism and structural properties that describe the morphology of the final material. A comparison of these quantities can give us the morphology to reaction mechanism dependency.

In order to characterize the chemical properties of the reaction process we focus on two quantities. First, we define the bond fraction ${\mathrm{BF}}_{\xi }$ of reaction center in a specific state ξ or of bond vector types ξ. ${\mathrm{BF}}_{\xi }$ is the ratio of the actual number and the maximal possible number of reaction centers in the specific state or of bond vector types. Second, we determine the ratio of the mean number of actual to initial nearest-neighbor contacts between beads of identical type ($\langle {\hat{n}}_{\alpha \alpha }\left(t_{\mathrm{fin}}\right)\rangle /\langle {\hat{n}}_{\alpha \alpha }\left(t_{\mathrm{ini}}\right)\rangle$) or different types ($\langle {\hat{n}}_{\alpha \beta }\left(t_{\mathrm{fin}}\right)\rangle /\langle {\hat{n}}_{\alpha \alpha }\left(t_{\mathrm{ini}}\right)\rangle$). Both quantities reflects the connectivity and network formation between the beads during the reaction process. Note in the following α and β are general replacements for different bead types. It can be either A, B, or A∪B.

To characterize the structural properties of the final rBFM morphologies, we determine the following structural properties

- the bulk porosity ${\bar{\Phi }}_{\alpha }$ and the specific surface area $S_{V_{\alpha }}$ with α={A,B,A∪B},
- the radial distribution function $g_{\beta \alpha }$ with βα={AA,BB} and the local porosity distribution $\mu_{\alpha }$ with α={A,B,A∪B}, as well as
- the percolation probability $\vartheta_{\alpha ,d}$ and the percolation fraction $\theta_{\alpha ,d}$ with α={A,B,A∪B} and *d* as listed in Table 2.

The bulk porosity ${\bar{\Phi }}_{\alpha }$ and specific surface area $S_{V_{\alpha }}$, i.e., surface area to volume ratio, are typical structural properties of porous materials. They give an insight whether the material has a large internal surface or volume, which is an important information for applications of the material for filter system, catalyst or gas storage. They are determined as

(3) $$\begin{array}{cc} {\bar{\Phi }}_{\alpha } & =\frac{V}{V_{\alpha }}\;\mathrm{and}\;S_{V_{\alpha }}=\frac{A_{\alpha }}{V_{\alpha }}, \end{array}$$

where $V=L^{3}$ is the total volume of L, $V_{\alpha }$ is the bulk volume of the α beads, and $A_{\alpha }$ is corresponding the surface area. Counting the number of α beads on the lattice gives $V_{\alpha }$ and $A_{\alpha }$ is obtained by counting the number of free surfaces of α beads, i.e., surfaces where the neighboring lattice site is not occupied by an α bead. As *V* of L and *N* are fixed per simulation run, the bulk porosities are known in advance. For the bead type A ${\bar{\Phi }}_{A}=1/6$, for the bead type B ${\bar{\Phi }}_{B}=1/3$, and for the overall material A∪B we have ${\bar{\Phi }}_{A\cup B}=1/2$.

To characterize the pore structure of the nanoporous hybrid material experimentally the pore size distribution is determined. In terms of numerical simulations typically the radial distribution function (RDF) $g_{\beta \alpha }$ and the local porosity distribution $\mu_{\alpha }$ [31] are evaluated, which can be related to the pore size distribution [9]. $g_{\beta \alpha }\left(r\right)$ characterizes typical radial distances of clusters of a bead type α to a centered β bead. This can be obtained by counting number of α beads $dn_{\beta \alpha }$ in a shell of width dr at a radial distance *r* from a centered β bead. The RDF $g_{\beta \alpha }$ is then given as

(4) $$\begin{array}{cc} g_{\beta \alpha }\left(r\right) & =\frac{dn_{\beta \alpha (r)}}{4\;\pi \;r^{2}\;\rho_{\alpha }\;dr}\;\mathrm{with}\;\rho_{\alpha }=N_{\alpha }/V, \end{array}$$

where the index βα={AA, BB}. Note that if all α beads are distributed homogenous $g_{\beta \alpha }\left(r\right)=1$.

The local porosity distribution $\mu_{\alpha }\left(\Phi_{\alpha },K\right)$ is an empirical probability density distribution that characterizes fluctuations of the porosity ${\bar{\Phi }}_{\alpha }$ in the material. It can be derived by defining measurement cells $K(\vec{x},K)$ at position $\vec{x}$ of size K×K×K with K<L and a corresponding total volume $\overset{ˇ}{V}$. These measurement cells are placed at *k* possible sites on the lattice L and then the local porosity distribution can be determined as

(5) $$\begin{array}{cc} \mu_{\alpha }\left(\Phi_{\alpha },K\right) & =\frac{1}{k}\sum_{\vec{x}}\delta \left(\Phi_{\alpha }-\Phi_{\alpha }\left(\vec{x},K\right)\right)\;\mathrm{with}\;\Phi_{\alpha }\left(\vec{x},K\right)=\overset{ˇ}{V}/{\overset{ˇ}{V}}_{\alpha } \end{array}$$

with the local porosity $\Phi_{\alpha }$,${\overset{ˇ}{V}}_{\alpha }$ being the volume of α beads in $\overset{ˇ}{V}$, and the δ-distribution.Note that we place K on lattice sites that have a distance of at least K/2 lattice sites from the boundary.

Additionally, we investigate the connectivity of the beads by analyzing fluctuations of the percolation probability $\vartheta_{\alpha ,d}$ and the total percolation fraction $\theta_{\alpha ,d}$ in direction *d* (see Table 2). A material is percolating, if there is a closed path of α beads from one side of K to the another side via direction *d*. The percolation probability is defined as

(6) $$\begin{array}{cc} \vartheta_{\alpha ,d}\left(\Theta_{\alpha ,d},K\right) & =\frac{\sum_{\vec{x}}\Theta_{\alpha ,d}\left(\vec{x},K\right)\;\delta_{\Phi_{\alpha }\Phi_{\alpha }\left(\vec{x},K\right)}}{\sum_{\vec{x}}\delta_{\Phi_{\alpha }\Phi_{\alpha }\left(\vec{x},K\right)}} \end{array}$$

with

(7) $$\begin{array}{cc} \Theta_{\alpha ,d}\left(\vec{x},K\right) & =\left\{\begin{array}{cc} 1, & \mathrm{if}\;K(\vec{x},K)\;\mathrm{peroclates}\;\mathrm{in}\;d\text{-}\mathrm{direction},\\ 0, & \mathrm{otherwise}. \end{array}\right. \end{array}$$

$\vartheta_{\alpha ,d}$ represents the fraction of measurement cells K of side length *K* with local porosity $\Phi_{\alpha }$ that are percolating in direction *d*. If d=x,y,z the corresponding space directions if d=3 in all directions at the same time, if d=c in at least one direction, and if d=0 no percolation at all is indicated.From this one can determine the total fraction of percolating cells as

(8) $$\begin{array}{cc} \theta_{\alpha ,d}\left(K\right) & =\int_{0}^{1}\mu_{\alpha }\left(\Phi_{\alpha },K\right)\;\vartheta_{\alpha ,d}\left(\Phi_{\alpha },K\right)\;d\Phi_{\alpha }, \end{array}$$

which is an important quantity to design a network representation of the morphology, as $\theta_{\alpha ,d}$ gives the fraction of network elements (bonds, sites, ⋯) that have to be permeable.

## 3. Results and Discussion

The model parameters under investigation and the analyzed valuesare specified in Table 1. We chose the parameter values in order to sample a broad parameter space close to a log-scale around the control parameter set, which reproduced the twin polymerization as shown in [9]. Thus, there are 288 different parameter combinations per system size *L*. In the following we discuss the influence of the different parameters and the occurring effects on the reaction mechanism, the structure formation process, and the morphology. During the analysis process, we first investigated and compared the single parameter combinations with each other. Thereby, typical values for the quantities under investigation are obtained for several parameter combinations and a clustering of the results suggested itself in order to reduce the complexity the results. We defined groups of parameter combinations, where certain parameter values are fixed, representing the typical clusters found.In Table 3 the notation and the corresponding fixed parameter value of the resulting 17 different parameter groups G(i) are given. For the analysis of the local porosity and the percolation we choose K={4,6,8,12,24,48} for the system size L=108 and smaller *K* for the other system sizes, respectively.

### 3.1. Analysis of the Model Parameters on the Reaction Process

#### 3.1.1. Bond Fraction

**Figure 4:**
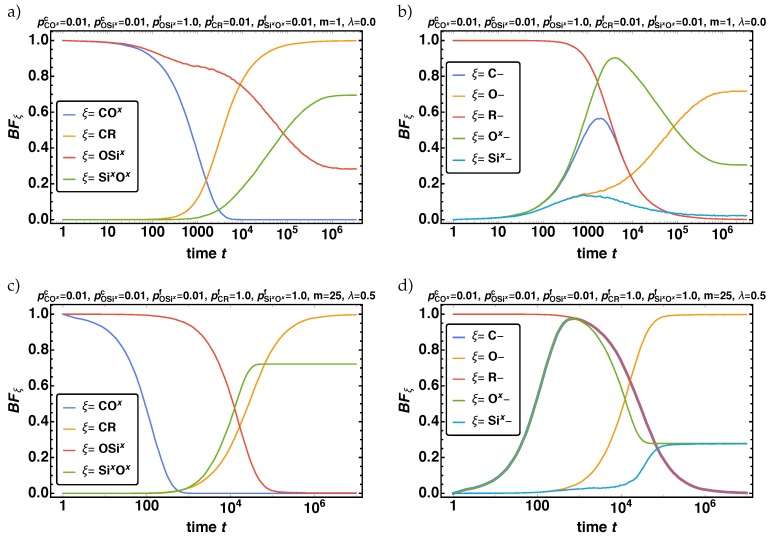
The time development of the bond fractions ${\mathrm{BF}}_{\xi }$ of all bond vector types ξ={CO*^x^*, OSi*^x^*, CR, Si*^x^*O*^x^*} in (**a**,**c**) and of the non-bonded reaction centers ξ={C–, O–, R–, O*^x^*–, Si*^x^*–} in (**b**,**d**) are given for the two exemplary parameter combinations (I) and (II) (**a**–**d**). Note that in (**d**) the curve for ξ=C– is plotted thicker, as it falls on top of the curve for R– and O*^x^*–.

First, we evaluate the influence of the model parameters on the time development of the overall reaction mechanism. With respect to the bond fraction ${\mathrm{BF}}_{\xi }$ we find a huge variety of time behaviors for the different reaction phases. In Figure 4, the time development of ${\mathrm{BF}}_{\xi }$ for all bond vector types and the non-bonded reaction centers are given over logarithmic time *t* for two exemplarily parameter combinations

(9) $$\begin{array}{cc} (I):\;p_{{\mathrm{CO}}^{x}}^{c} & =p_{\mathrm{CR}}^{f}=p_{{\mathrm{OSi}}^{x}}^{c}=p_{{\mathrm{Si}}^{x}O^{x}}^{f}=0.01,p_{{\mathrm{OSi}}^{c}}^{f}=1.0,m=1,\lambda =0.0,\;\mathrm{and} \end{array}$$

(10) $$\begin{array}{cc} (\mathrm{II}):\;p_{{\mathrm{CO}}^{x}}^{c} & =p_{{\mathrm{OSi}}^{x}}^{c}=p_{{\mathrm{Si}}^{x}O^{x}}^{f}=0.01,p_{\mathrm{CR}}^{f}=p_{{\mathrm{OSi}}^{c}}^{f}=1.0,m=25,\lambda =0.5. \end{array}$$

An exemplary initial and final 3D structure of a simulation run for the parameter combination (I) is shown in Figure 2.

For the combination (I) (Figure 4a,b) all CO*^x^* bond vectors cleave at first and then subsequent most of the OSi*^x^* bond vectors. The available non-bonded reaction centers allow then to first form a nearly complete network of A-beads via CR bond vector formation and then to some extend the B-bead network via Si*^x^*O*^x^* bond vectors grow. For the combination (II) (Figure 4c,d) first the bond vectors of type CO*^x^* and OSi*^x^* cleave completely. In this case first the B-bead network forms and second the A-bead network, contrary to (I) (compare Figure 4a,c). This has a drastical influence on ${\mathrm{BF}}_{O-},{\mathrm{BF}}_{O^{x}-},$ and ${\mathrm{BF}}_{{\mathrm{Si}}^{x}-}$ (compare Figure 4b,d).

Overall, we find a complex behavior of the reaction mechanism due to the different reaction steps that influence each other for the 288 parameter combinations. In detail it can be observed that:

- With decreasing *m* all reactions are shifted to later times.
- With decreasing values of λ a minor time shift to later times occur for reactions, where the non-bonded reaction centers O–, O*^x^*–, Si*^x^*–, and the bond vector types OSi*^x^*, Si*^x^*O*^x^* are involved. Additionally, we find different final values of ${\mathrm{BF}}_{\xi }$ depending on λ for ξ={O–, Si,– Si*^x^*O*^x^*}.
- With increasing $p_{{\mathrm{CO}}^{x}}^{c}$ the reactions, where C, R, CR, and CO*^x^* are related, are shifted to significantly shorter times, whereas the other processes are only influenced in a minor way.
- With decreasing $p_{\mathrm{CR}}^{f}$ the reaction, where C–, R–, and CR participate, are shifted to significantly later times. The other process steps are not affected.
- The parameter group $G_{{\mathrm{OSi}}^{x}}$ influences reactions in various ways, which are connected with O–, O*^x^*–, Si*^x^*–, OSi*^x^*, and Si*^x^*O*^x^*. In principle these parameter combinations change the duration of the reaction process, so that one can order it by increasing reaction duration. Ordering the results leads to $G_{{\mathrm{OSi}}^{x}}\left(3\right)<G_{{\mathrm{OSi}}^{x}}\left(4\right)<G_{{\mathrm{OSi}}^{x}}\left(1\right)<G_{{\mathrm{OSi}}^{x}}\left(2\right)$ with increasing duration. Similar to λ, here the influence on the reaction times shows up in the final values of ${\mathrm{BF}}_{\xi }$ for ξ={O–, Si*^x^*–, Si*^x^*O*^x^*}.
- With decreasing $p_{{\mathrm{Si}}^{x}O^{x}}^{f}$ reactions are shifted to later times, where O–, O*^x^*–, Si*^x^*–, OSi*^x^*, and Si*^x^*O*^x^* are involved.

Despite the influence of $p_{{\mathrm{CO}}^{x}}^{c}$, all effects are reflected by the parameter combination (I) and (II) shown in Figure 4.

From the above list, we can sum up that all parameters influence the time behavior of the reaction mechanism, but it seems that only some effects survive as a change in the final values of the bond fractions till the end of the reaction process and thus influence the final morphology. So we examine these changes in the bond fractions ${\mathrm{BF}}_{\xi }$ for $t_{\mathrm{fin}}$ depending on parameter groups G(i) (see Table 3). For each parameter combination we get one value per bond fraction per reaction center. leading to a histogram per bond fraction per reaction center containing 288 values. In order to visualize the effects of the different parameters combinations, we highlighted all histogram counts that are counted among a sub group *i* of a special parameter group $G{}_{x}\left(i\right)$ in a different color. Additionally, we determined the averaged bond fraction $\bar{{\mathrm{BF}}_{\xi }}$ per reaction center ξ for each parameter group G(i) as the arithmetic mean of all corresponding values. Note that also for the following quantities under investigation the arithmetic mean is used to determine the corresponding average.

**Figure 5:**
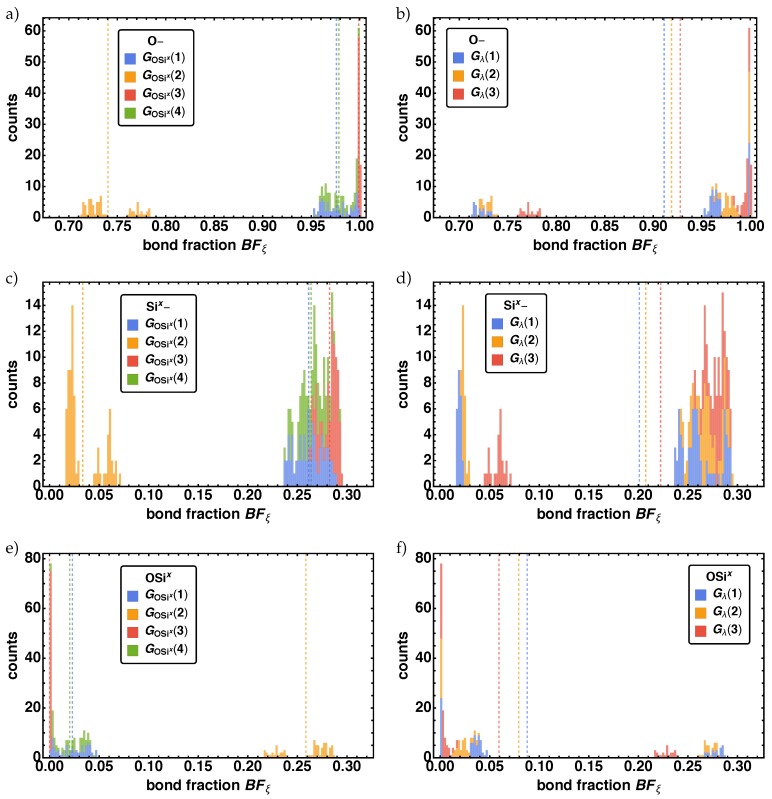
Distributions of the bond fractions ${\mathrm{BF}}_{\xi }$ at $t_{\mathrm{fin}}$ for the non-bonded reactions centers ξ={O–, Si*^x^*–} in (**a**–**d**), and the bond vector type ξ=OSi*^x^* in (**e**,**f**) for the two parameter combinations $G_{{\mathrm{OSi}}^{x}}$ (left column) and $G_{\lambda }$ (right column).

In Figure 5 the resulting histograms for the ${\mathrm{BF}}_{\xi }$ for ξ={O–, Si*^x^*–, OSi*^x^*} for the parameter groups $G_{\lambda }$ (left column) and $G_{{\mathrm{OSi}}^{x}}$ (right column) are given. Note that the vertical dashed lines indicate the averaged bond fraction $\bar{{\mathrm{BF}}_{\xi }}$ of the corresponding parameter groups G(i). For all three cases ξ={O–, Si*^x^*–, OSi*^x^*} each distribution exhibits one bigger (SD1) and two smaller subdistributions (SD2, SD3). For ${\mathrm{BF}}_{O-}$ SD1 is at (0.95,1.0], SD2 af (0.75,0.8), and SD3 at (0.7,0.75), which can be identified analog for ${\mathrm{BF}}_{{\mathrm{Si}}^{x}-}$ and ${\mathrm{BF}}_{{\mathrm{OSi}}^{x}}$, whereas for ${\mathrm{BF}}_{{\mathrm{OSi}}^{x}}$ the order is reversed. The two smaller subdistributions (SD2, SD3) are fully characterized by $G_{{\mathrm{OSi}}^{x}}\left(2\right)$, where the values of the bond fraction are significantly smaller for O– and Si*^x^*– (larger for OSi*^x^*) than the values of SD1. SD1 is characterized by $G_{{\mathrm{OSi}}^{x}}\left(1\right),G_{{\mathrm{OSi}}^{x}}\left(3\right)$, and $G_{{\mathrm{OSi}}^{x}}\left(4\right)$, where one observes an internal ordering with the smallest (largest) bond fraction values for $G_{{\mathrm{OSi}}^{x}}\left(1\right)$ and the largest (smallest) bond fraction values for $G_{{\mathrm{OSi}}^{x}}\left(3\right)$ for O and Si*^x^* (OSi*^x^*). Coloring the same bond fraction distribution with respect to $G_{\lambda }$, we find for ${\mathrm{BF}}_{O-}$ and ${\mathrm{BF}}_{{\mathrm{Si}}^{x}-}$ that SD1 has contributions of all $G_{\lambda }\left(i\right)$, with increasing (decreasing) order of *i*, i.e., $G_{\lambda }\left(1\right)<G_{\lambda }\left(2\right)<G_{\lambda }\left(3\right)$. The same order is found for SD2 and SD3, however there SD2 is completely described by $G_{\lambda }\left(3\right)$ and SD2 by $G_{\lambda }\left(1\right)<G_{\lambda }\left(2\right)$. Note that for ${\mathrm{BF}}_{{\mathrm{OSi}}^{x}}$ the same effect but reversed order is found.

**Figure 6:**
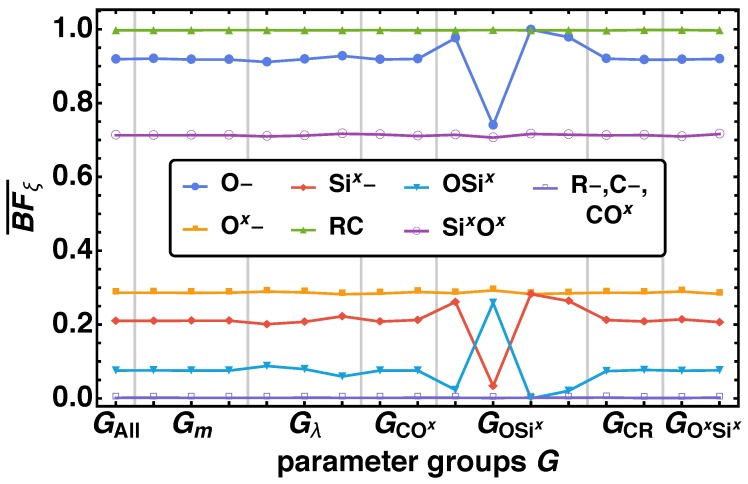
Averaged bond fractions $\bar{{\mathrm{BF}}_{\xi }}$ at $t_{\mathrm{fin}}$ for all non-bonded reaction center ξ={O–, C–, R–, O*^x^*–, Si*^x^*–} and bond vector types ξ={CO*^x^*, CR, OSi*^x^*, Si*^x^*O*^x^*} over the parameter groups G(i) specified in Table 3.

The same behavior is also reflected by the averaged bond fractions $\bar{{\mathrm{BF}}_{\xi }}$ over the parameter groups G(i) as shown in Figure 6. $G\left(1\right)=G_{\mathrm{all}}$ represents the averaged bond fraction over all parameter combinations. We observe variations of $\bar{{\mathrm{BF}}_{\xi }}$ over G(i) with i>1 from the overall $\bar{{\mathrm{BF}}_{\xi }}$ of $G_{\mathrm{all}}$ for ξ={O–, Si*^x^*–, OSi*^x^*} for two parameter groups $G_{{\mathrm{OSi}}^{x}}$ and $G_{\lambda }$, where the effect of OSi*^x^* is inverse to O– and Si*^x^*–. There is a significant variation for $G_{{\mathrm{OSi}}^{x}}\left(i\right)$, where we can order the parameter groups with decreasing (increasing) order of $\bar{{\mathrm{BF}}_{\xi }}$ of O– and Si*^x^*– (OSi*^x^*) as $G_{{\mathrm{OSi}}^{x}}\left(3\right)<G_{{\mathrm{OSi}}^{x}}\left(4\right)<G_{{\mathrm{OSi}}^{x}}\left(1\right)<G_{{\mathrm{OSi}}^{x}}\left(2\right)$, which is also derived from the distributions above. Furthermore, we find the same minor increase (decrease) of ${\mathrm{BF}}_{\xi }$ for the parameter combinations $G_{\lambda }\left(1\right)<G_{\lambda }\left(2\right)<G_{\lambda }\left(3\right)$ for O– and Si*^x^*– (OSi*^x^*) as from the distributions itself.

#### 3.1.2. Phase Separation

**Figure 7:**
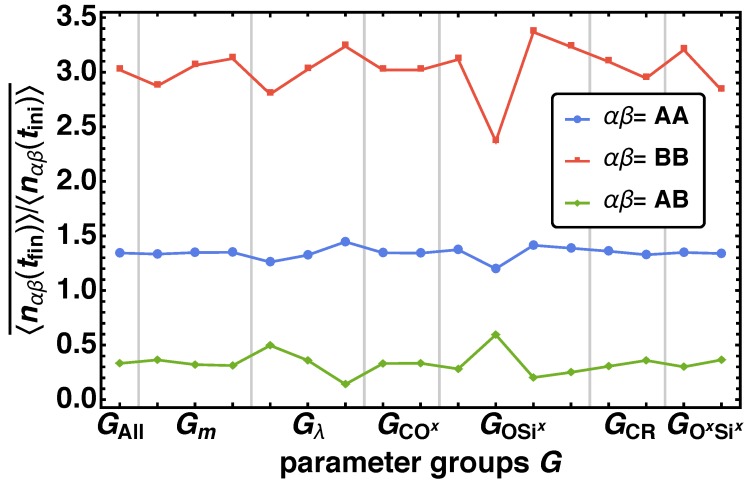
Averaged mean nearest-neighbor contacts $\bar{\langle {\hat{n}}_{\alpha \beta }\left(t_{\mathrm{fin}}\right)\rangle /\langle {\hat{n}}_{\alpha \beta }\left(t_{\mathrm{ini}}\right)\rangle }$ for all parameter groups G(i) for the bead type combinations αβ={AA, AB, BB}.

In the next step we investigate the phase separation behavior of the reaction mechanism depending on G(i). Again, for each parameter combination we obtain one value respectively for the mean nearest-neighbor contacts $\langle {\hat{n}}_{\alpha \beta }\left(t_{\mathrm{fin}}\right)\rangle /\langle {\hat{n}}_{\alpha \beta }\left(t_{\mathrm{ini}}\right)\rangle$ with αβ being the bead type combinations AA, AB, and BB. We determine the averaged mean nearest-neighbor contacts $\bar{\langle {\hat{n}}_{\alpha \beta }\left(t_{\mathrm{fin}}\right)\rangle /\langle {\hat{n}}_{\alpha \beta }\left(t_{\mathrm{ini}}\right)\rangle }$ as the arithmetic mean of all values that belong to the parameter combination G(i). In Figure 7 $\bar{\langle {\hat{n}}_{\alpha \beta }\left(t_{\mathrm{fin}}\right)\rangle /\langle {\hat{n}}_{\alpha \beta }\left(t_{\mathrm{ini}}\right)\rangle }$ over the parameter groups G(i) (see Table 3) are depict. We observe a more complex behavior for $\bar{\langle {\hat{n}}_{\alpha \beta }\left(t_{\mathrm{fin}}\right)\rangle /\langle {\hat{n}}_{\alpha \beta }\left(t_{\mathrm{ini}}\right)\rangle }$ than for $\bar{{\mathrm{BF}}_{\xi }}$ (compare Figure 6). Again a significant change of $\bar{\langle {\hat{n}}_{\alpha \beta }\left(t_{\mathrm{fin}}\right)\rangle /\langle {\hat{n}}_{\alpha \beta }\left(t_{\mathrm{ini}}\right)\rangle }$ for $G_{{\mathrm{OSi}}^{x}}$ and $G_{\lambda }$ is found. Additionally, deviations for $G_{m}$, $G_{\mathrm{CR}}$, and $G_{{\mathrm{Si}}^{x}O^{x}}$ for BB in major and for ABin minor occurrence can be identified. We find that with increasing *m* decreasing $p_{\mathrm{CR}}^{f}$ and $p_{{\mathrm{Si}}^{x}O^{x}}^{f}$ the relative number mean nearest-neighbor contacts increases, whereas the effect is inverted for AB. This means that the reduced movability and low network formation reactions hinder the formation of clusters, indicated by a high number of nearest neighbor contact.

From the above analysis of the time development of the reaction process we can conclude that if highly interweaved material with high internal connectivity and no phase separation is need, the twin monomers need to fulfill two important features: A and B should be as miscible as possible (λ low) and the movability should be low, while one of the connecting bonds has to have a high activation energy ($G_{{\mathrm{OSi}}^{x}}\left(2\right)$) to compensate phase separation and the corresponding formation of larger cluster.

### 3.2. Analysis of the Model Parameters on the Structure

In the following, we present the results for the structure properties of the overall material A∪B, as well as for the subsystems A and B. We analyze the specific surface area $S_{V_{\alpha }}$, the local porosity distribution $\mu_{\alpha }$, the percolation probability $\vartheta_{\alpha ,d}$, and the percolation fraction $\theta_{\alpha ,d}$ with α={A,B,A∪B}. and the RDF $g_{\alpha \beta }$ with αβ={AA, BB}. As in the previous section, next to the quantities itself we also look at the arithmetic means $\bar{\left(\cdot \right)}$, i.e., called averaged values in the following, over G(i), if possible.

#### 3.2.1. Bulk Porosity and Specific Surface Area

**Figure 8:**
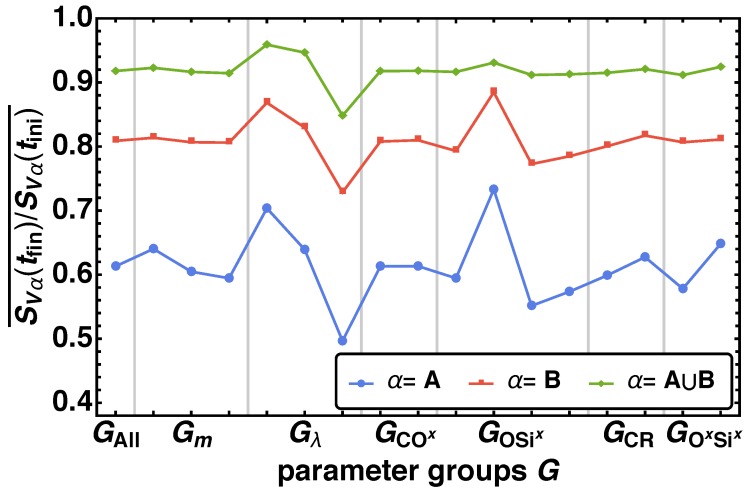
The averaged relative specific surface area $\bar{S_{V_{\alpha }}\left(t_{\mathrm{fin}}\right)/S_{V_{\alpha }}\left(t_{\mathrm{ini}}\right)}$ is given for the parameter groups G(i) (see Table 3).

In Section 2.4 the bulk porosities for our system are given as ${\bar{\Phi }}_{A}=1/6$, ${\bar{\Phi }}_{B}=1/3$, and ${\bar{\Phi }}_{A\cup B}=1/2$. Looking at the specific surface areas $S_{V_{\alpha }}$ for α={A,B,A∪B} we find significant changes of the averaged relative specific surface area $\bar{S_{V_{\alpha }}\left(t_{\mathrm{fin}}\right)/S_{V_{\alpha }}\left(t_{\mathrm{ini}}\right)}$. In Figure 8 the averaged relative specific surface areas $\bar{S_{V_{\alpha }}\left(t_{\mathrm{fin}}\right)/S_{V_{\alpha }}\left(t_{\mathrm{ini}}\right)}$ are given over the parameter groups G(i). In the case of A∪B the strongest influence factor is the attraction parameter λ, where one observes a decrease of $\bar{S_{V_{A\cup B}}\left(t_{\mathrm{fin}}\right)/S_{V_{A\cup B}}\left(t_{\mathrm{ini}}\right)}$ with increasing larger λ. For $G_{{\mathrm{OSi}}^{x}}$ and $G_{{\mathrm{Si}}^{x}O^{x}}$ a minimal change in $\bar{S_{V_{A\cup B}}\left(t_{\mathrm{fin}}\right)/S_{V_{A\cup B}}\left(t_{\mathrm{ini}}\right)}$ is observable. These effects are magnified for the subsystems A and B. We find an increasing $\bar{S_{V_{\alpha }}\left(t_{\mathrm{fin}}\right)/S_{V_{\alpha }}\left(t_{\mathrm{ini}}\right)}$ for the parameter groups in the order $G_{{\mathrm{OSi}}^{x}}\left(3\right)<G_{{\mathrm{OSi}}^{x}}\left(4\right)<G_{{\mathrm{OSi}}^{x}}\left(1\right)<G_{{\mathrm{OSi}}^{x}}\left(2\right)$, where the difference between $G_{{\mathrm{OSi}}^{x}}\left(2\right)$ and the other three groups is in same order as between $G_{\lambda }\left(1\right)$ and $G_{\lambda }\left(3\right)$. Additionally, we find a secondary effect for the parameter groups $G_{m},G_{\mathrm{CR}},$ and $G_{{\mathrm{Si}}^{x}O^{x}}$. There an increase of $\bar{S_{V_{\alpha }}\left(t_{\mathrm{fin}}\right)/S_{V_{\alpha }}\left(t_{\mathrm{ini}}\right)}$ for α={A, B} for decreasing *m* and increasing values of $p_{\mathrm{CR}}^{f}$ and $p_{{\mathrm{Si}}^{x}O^{x}}^{f}$ is recognized.

Similar to the bond fraction, and nearest-neighbor analysis we find that nanoporous material with a high specific surface area are obtained, when the twin monomer is designed of components A and B that are are miscible (low λ) and the energy barrier of the final connecting bond is high ($G_{{\mathrm{OSi}}^{x}}\left(2\right)$). Furthermore, a low movability (low *m*) and a prefered and fast bond formation between identical bead types (high $p_{\mathrm{CR}}^{f}$ and $p_{{\mathrm{Si}}^{x}O^{x}}^{f}$) support a high specific surface area of the material.

#### 3.2.2. Radial Distribution Function

**Figure 9:**
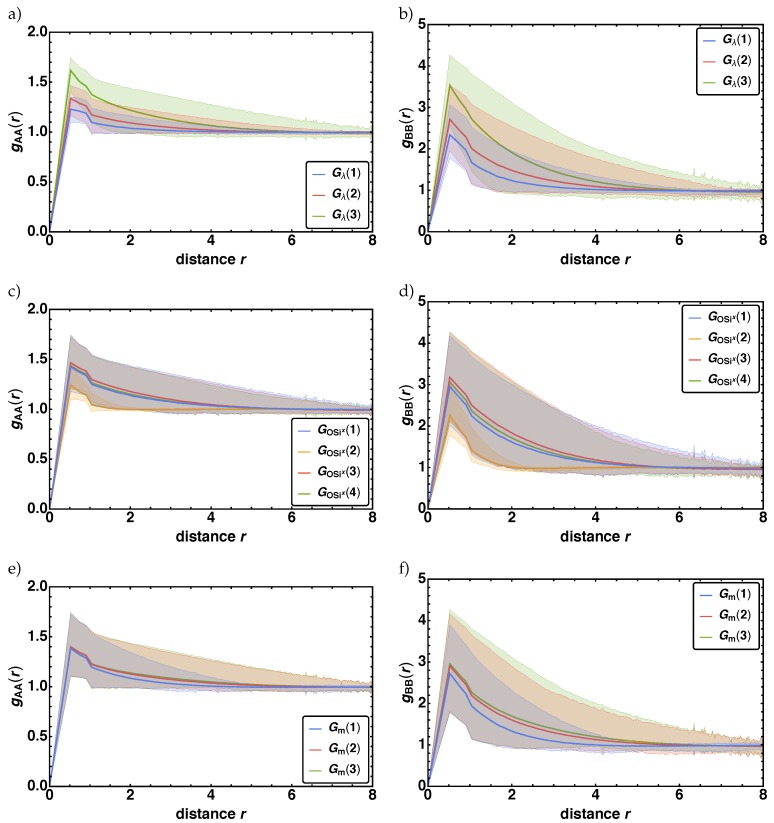
The arithmetic mean, i.e., averaged (thick colored line), the minimum (lowest thin colored line) and the maximum (highest thin colored line) radial distribution function $g_{\beta \alpha }\left(r\right)$ over radial distance *r* are given for ba = fAA, BBg in (**a**,**c**,**e**) and (**b**,**d**,**f**) and for the parameter groups $G_{\lambda },G_{{\mathrm{OSi}}^{x}}$, and $G_{m}$ in (**a**,**b**), (**c**,**d**), and (**e**,**f**). Each subgroup i is highlighted with a different color.

The analysis of the RDF $g_{\beta \alpha }\left(r\right)$, i.e., per each parameter combination there is one resulting RDF, exhibited that the influence of $p_{{\mathrm{CO}}^{x}}^{c}$ is neglectable and an increase of $p_{\mathrm{CR}}^{f}$ leads to a minor increase of $g_{\mathrm{AA}}$ and $p_{{\mathrm{Si}}^{x}O^{x}}^{f}$ of $g_{\mathrm{BB}}$. The main effects are observed for the parameter groups $G_{\lambda }$ (a)-b)), $G_{{\mathrm{OSi}}^{x}}$ (c)-d)), and $G_{m}$ (e)-f)), as depicted in Figure 9. Here, we present the arithmetic mean RDF $g_{\beta \alpha }$ of all RDFs per parameter combinations that belong to the G(i) (thick colored line), as well as the minimum (lowest thin colored line) and maximum (highest thin colored line) $g_{\beta \alpha }\left(r\right)$ out of the corresponding parameter group $G{}_{x}\left(i\right)$. The area between the minimum and the maximum curve is covered by the various parameter combination within each parameter group and colored in the same color. In the left column the results for βα=AA and in the right column for BB are presented. Note, that the corresponding subgroups *i* of each parameter group $G{}_{x}\left(i\right)$ are highlighted in different colors. From the plots we find two parameter groups with major variations and one with minor one. The major effects are again observed for λ and for $G_{{\mathrm{OSi}}^{x}}$. With increasing λ $g_{\mathrm{AA}}\left(r\right)$ and $g_{\mathrm{BB}}\left(r\right)$ gets higher and wider by a factor of around 1.5–2 in height and width. For BB additionally we observe that for small values of λ the maximum is truncated. For $G_{{\mathrm{OSi}}^{x}}$ a major difference is observed between $G_{{\mathrm{OSi}}^{x}}\left(2\right)$ and $G_{{\mathrm{OSi}}^{x}}\left(i\right)$ with i=1,3,4. The distributions for $G_{{\mathrm{OSi}}^{x}}\left(2\right)$ are narrow and spiky with lower maximal values, whereas the distributions of the other three groups are broader with higher values, depending on λ as well. Note that for $G_{{\mathrm{OSi}}^{x}}$ the width increases up to a factor of 5, whereas the height increases by factor of 1.5–2. Minor difference is displayed by $G_{m}$, where find that with increasing *m* the RDF $g_{\beta \alpha }\left(r\right)$ primary gets wider (factor around 1.5) and only a minimal increase in height.

#### 3.2.3. Local Porosity Distribution

**Figure 10:**
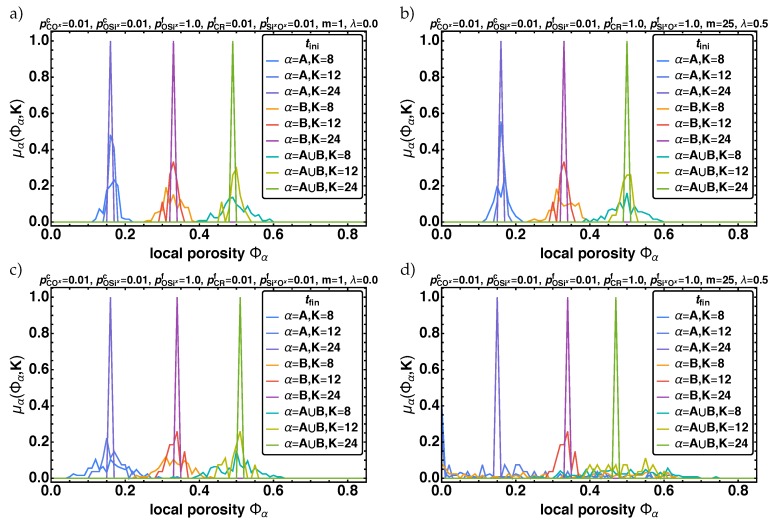
The local porosity distributions $\mu_{\alpha }\left(\Phi_{\alpha },K\right)$ for K={8,12,24} and α={A, B, A∪B} are given over the local porosity $\Phi_{\alpha }$ for the two parameter combinations (I) and (II). In (**a**,**b**) the initial and in (**c**,**d**) the final distributions are shown.

**Figure 11:**
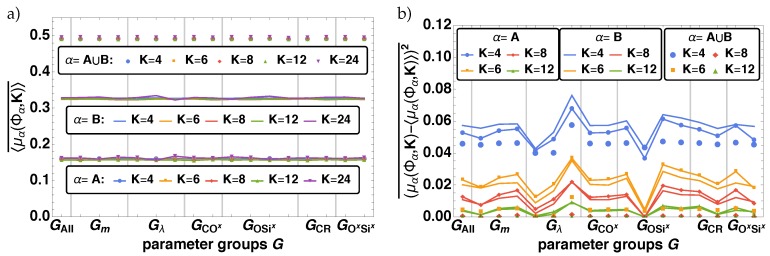
In a) the averaged mean values of the local porosity distribution $\bar{\langle \mu_{\alpha }\left(\Phi_{\alpha },K\right)\rangle }$ and in b) the averaged variances $\bar{{\left(\mu_{\alpha }\left(\Phi_{\alpha },K\right)-\langle \mu_{\alpha }\left(\Phi_{\alpha },K\right)\rangle \right)}^{2}}$ for different lengths K={4,6,8,12} of the measurement cells for α={A, B, A∪B} are shown over the parameter groups G(i). Note that for K>12 all variances are 0 and thus are not shown here.

Alternatively to the RDF, the local porosity distribution $\mu_{\alpha }$ is used to characterize the pore structure of the material. In Figure 10 the results for the local porosity distributions $\mu_{\alpha }\left(\Phi_{\alpha },K\right)$ for α={A,B,A∪B} are shown exemplary for K={8,12,24} at $t_{\mathrm{ini}}$ in (a,b), and $t_{\mathrm{fin}}$ in (c,d) for the two parameter combinations (I) and (II) given in Equations (9) and (10). Analyzing the distributions depending on *K*, we observe that in all cases the distributions get localized around ${\bar{\Phi }}_{\alpha }$. This is accepted, as $\mu_{\alpha }$ represents the fluctuations of $\Phi_{\alpha }$ for *K* and when K→L follows that $\Phi_{\alpha }\to {\bar{\Phi }}_{\alpha }$ [31]. Furthermore, we find that the initial distributions for α={A,B,A∪B} for (I) and (II) are similar in shape independed of the parameters. This reflects the random initialization of the beads on the lattice. However for $t_{\mathrm{fin}}$ the distributions are quite different. For (I) the final distributions is more narrow and peaked (see Figure 10c), whereas for (II) they get wider and flatter (see Figure 10d). As the changing width is an important feature of these distributions we will also analyze the variance ${\left(\mu_{\alpha }\left(\Phi_{\alpha },K\right)-\langle \mu_{\alpha }\left(\Phi_{\alpha },K\right)\rangle \right)}^{2}$ of the local porosity distributions, next to their mean values $\langle \mu_{\alpha }\left(\Phi_{\alpha },K\right)\rangle$ (Figure 11).

The averaged mean values of the local porosity distributions $\bar{\langle \mu_{\alpha }\left(\Phi_{\alpha ,K}\right)\rangle }$ are nearly constant at values of the corresponding bulk porosity ${\bar{\Phi }}_{\alpha }$ (see Figure 11a). The variances however, display the previous findings, i.e., changes depending on the parameter groups $G_{m},G_{\lambda },G_{{\mathrm{OSi}}^{x}},G_{\mathrm{CR}},$ and $G_{{\mathrm{Si}}^{x}O^{x}}$. In Figure 10b the averaged variances $\bar{{\left(\mu_{\alpha }\left(\Phi_{\alpha },K\right)-\langle \mu_{\alpha }\left(\Phi_{\alpha },K\right)\rangle \right)}^{2}}$ are given over the parameter groups G(i) (see Table 3). We observe for smaller measurement cells larger the fluctuations. For K>6 in the case of α=A∪B and for K>12 for the subsystems the variances goes down to 0, thus only results for K≤12 are plotted. The observed changes in the variance fit very well with the previous findings. The major impact is obtained by varying $G_{\lambda }$ and $G_{{\mathrm{OSi}}^{x}}$. For all α the variance increases for $G_{\lambda }\left(i\right)$ with increasing *i* and we observe the smallest variance for $G_{{\mathrm{OSi}}^{x}}\left(2\right)$ and the largest for $G_{{\mathrm{OSi}}^{x}}\left(3\right)$. This means the more attractive the beads the wider gets the distribution and the more clusters of different sizes are found. This effect is enhanced if the final connecting bond has a low energy barrier and beads separate easily. Furthermore, the subsystems A and B show a minor dependency on $G_{m},G_{\mathrm{CR}}$, and $G_{{\mathrm{Si}}^{x}O^{x}}$. We find a decrease of $\bar{{\left(\mu_{\alpha }\left(\Phi_{\alpha },K\right)-\langle \mu_{\alpha }\left(\Phi_{\alpha },K\right)\rangle \right)}^{2}}$ for decreasing *m* or for increasing $p_{\mathrm{CR}}^{f}$ and $p_{{\mathrm{Si}}^{x}O^{x}}^{f}$, i.e., the less the beads move and the faster the networks are formed the more homogenous are the cluster sizes in the final material.

#### 3.2.4. Percolation

**Figure 12:**
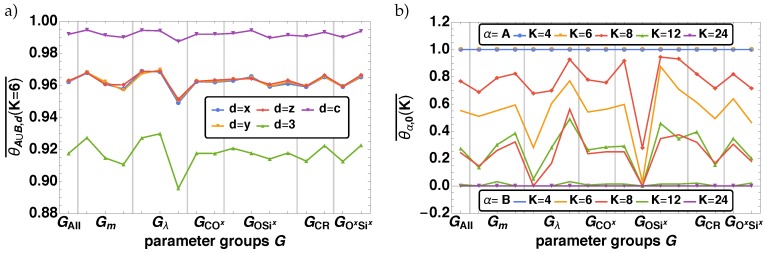
The averaged total fraction of percolating measurement cells $\theta_{\alpha ,d}\left(K\right)$ with $\theta_{\alpha ,d}\left(K\right)\neq 0$ and 1 are given over the parameter groups G(i) (see Table 3). The results are shown in (**a**) for α=A∪B, K=6, and d={x,y,z,3,c}, whereas in (**b**) for α={A, B} and d=0.

Finally, we investigate the total fraction of percolating measurement cells along different directions *d* given in Table 2 for different cell sizes *K*. We find that the obtained structures are fully percolating or not at all in nearly all cases, i.e., $\theta_{\alpha ,d}\left(K\right)=1$ for d={x,y,z,3,c} and 0 for d=0. There are two exceptions: α=A∪B and d={x,y,z,3,c} at small K={6,8} and α={A, B} and d=0. These two cases are shown in Figure 12a,b. As the effects for K={6,8} are similar, but the magnitude of the effect for K=6 is larger, we present here only the results for K=6.

In Figure 12a the averaged total fraction of percolating measurement cells $\bar{\theta_{\alpha ,d}\left(K\right)}$ is shown for d≠0 and α=A∪B at K=6. We find an overall high value of $\bar{\theta_{A\cup B,d}\left(6\right)}$. The highest value is observed for d=c, i.e., there is nearly always a percolating direction within the material. The second highest $\bar{\theta_{A\cup B,d}\left(6\right)}$ are found for any single percolating direction (d=x,y,z). Here all three directions are nearly the same, thus there is no biased direction within the material. The lowest value is obtained for a percolation all three directions at the same time, i.e., d=c. Independent of the percolating direction, we find an decreasing $\bar{\theta_{\alpha ,d}\left(6\right)}$ for increasing λ, as the beads form packed clusters that are not necessarily connected to both sides of the measurement cell and thus can not percolate. We find that an enhanced network formation (high value of $p_{\mathrm{CR}}^{f},p_{{\mathrm{Si}}^{x}O^{x}}^{f}$) and a low movability of the beads (low value of *m*), increases $\bar{\theta_{A\cup B,d}\left(K\right)}$. This can be accounted to the high connectivity of identical beads due to the fast network formation or due to the homogeneous distribution of beads, as they can not move too far away from the initial homogeneous distribution. Interestingly, $G_{{\mathrm{OSi}}^{x}}$ shows here only a minor influence on the percolation for A∪B. In Figure 12b $\bar{\theta_{\alpha ,0}\left(K\right)}$ we show for A and B for all *K*. Here, the case of no percolation in any direction is analyzed. As mentioned above, for $\bar{\theta_{A\cup B,0}\left(K\right)}=0$, but for the subsystems it is not. Analyzing the general behavior of $\bar{\theta_{\alpha ,0}\left(K\right)}$ over *K* we find that for the bead type A that percolation is restored for system size K>12, whereas for B it already restored for K>8. For both subsystems we find changes in $\bar{\theta_{\alpha ,0}\left(K\right)}$, depending on $G_{\lambda },G_{{\mathrm{OSi}}^{x}},G_{\mathrm{CR}},G_{{\mathrm{Si}}^{x}O^{x}},$ and $G_{\lambda }$, similar to the previous results. With increasing λ $\bar{\theta_{\alpha ,0}\left(K\right)}$ decreases, as the probability of percolation increases. There is a drastic drop of $\bar{\theta_{\alpha ,0}\left(K\right)}$ for $G_{{\mathrm{OSi}}^{x}}\left(2\right)$, as the high connectivity of the beads for this parameter combination typically leads to percolation. This effect is also indicated by the decrease of $\bar{\theta_{\alpha ,0}\left(K\right)}$ for increasing $p_{\mathrm{CR}}^{f},p_{{\mathrm{Si}}^{x}O^{x}}^{f}$. Finally the decrease of $\bar{\theta_{\alpha ,0}\left(K\right)}$ with decreasing *m* is due to the low movability that keeps the beads close to their initial random starting position on the lattice, which enhances the chance of percolation.

## 4. Summary

The focus of this work was the derivation of a structure on reaction process dependency for twin polymerization and the identification of influencing factors to modify the final morphology to allow an application-oriented adaptation of the final twin polymers. Therefore, we utilized the previously introduced reactive bond fluctuation model for twin polymerization of the typical twin monomer 2,2${}^{\prime }$-spiro[4*H*-1,3,2-benzodioxasiline]. We kept the reaction mechanism and the basic composition of the twin monomer for our analysis as both can be recovered for many other twin monomers as well. However, we varied all reaction relevant parameters and analyzed their effects on the final morphology. In this context, we investigate various chemical and structural properties, that are directly or indirectly connected with the reaction mechanism, as the bond fractions, the specific surface area, the bulk and the local porosity, percolation effects and the radial distribution function for a wide range of possible parameter combinations.

Our investigations showed that there are four main components that affect the final morphologies; most of all: That are the attraction between the two chemical components (beads), the strength, i.e., activation energy, of the final connecting bond between two different bead types, the probability of identical beads to form bonds and the movability of the beads. The more attractive identical bead types are the more often the beads are bonded and thus the larger and more heterogenous are the clusters sizes of identical beads. Consequently, the specific surface area decreases, the cluster size and number of beads per cluster increases, and the percolation probability decreases. The higher activation energy of the final connecting bond, the longer are the beads of different type connected, and thus, the smaller are the cluster sizes and beads per cluster leading to more homogenous materials. This is also reflected in the percolation property of the obtained materials, as a high connectivity increases the probability to find percolation. The described effects of the strength of the final connecting bond can be enhanced by increasing the probability to form networks between identical bead types. Additionally, we found that the movability influences the structural properties of the material as well. The higher the movability, the larger the cluster sizes and number of beads per clusters, i.e., the material is more heterogenous due to the different cluster sizes and this decreases the percolation probability, however, note that the bond fractions have not been affected by the movability.

Hence, we found a direct dependency of the reaction relevant parameters to structural properties of the final morphology. We were able to deduce direct proportionalities on how to change the underlying components to design certain effects, i.e., a high specific surface area or homogeneous cluster sizes, within the material. At this point it is now of special interest to analyze the already known class of twin monomers with respect to the above-mentioned main components that affect the morphology and to compare the resulting findings with the properties of the used 2,2${}^{\prime }$-spiro[4*H*-1,3,2-benzodioxasiline]. So we will be able to classify the twin monomers within our parameter groups and to check our prediction on the real materials.

## Figures and Tables

**Table 1:** All varied model parameters and corresponding analyzed values of the reactive bond fluctuation model are listed.

Model Parameter (Variable)	Symbol	Analyzed Values
CO*^x^* cleavage	$p_{{\mathrm{CO}}^{x}}^{c}$	0.01,1.0
OSi*^x^* cleavage	$p_{{\mathrm{OSi}}^{x}}^{c}$	0.01,1.0
OSi*^x^* formation	$p_{{\mathrm{OSi}}^{x}}^{f}$	0.01,1.0
CR formation	$p_{\mathrm{CR}}^{f}$	0.01,1.0
Si*^x^*O*^x^* formation	$p_{{\mathrm{Si}}^{x}O^{x}}^{f}$	0.01,1.0
ratio rMCS/nMCS	*m*	1,10,25
attraction parameter	λ	0.0,0.1,0.5

**Table 2:** Investigated percolation directions *d*. With index d={x,y,z} the corresponding space directions are indicated. Index d=3 represent percolation in all directions at the same time, d=c at least one direction, and d=0 stands for no percolation at all.

Index *d*	Meaning	Index *d*	Meaning
*x*	*x*-direction	3	(x∧y∧z)-direction
*y*	*y*-direction	*c*	(x∨y∨z)-direction
*z*	*z*-direction	0	!(x∨y∨z)-direction

**Table 3:** 17 parameter groups G(i) are defined, where typically one parameter value is fixed. The corresponding parameter values for each group are given in first column and the short notation in the second.

Model Parameter	Notation	Model Parameter	Notation
average over all	$G\left(1\right)=G_{\mathrm{all}}$	$p_{{\mathrm{OSi}}^{x}}^{c}=0.01,p_{{\mathrm{OSi}}^{x}}^{f}=0.01$	$G\left(10\right)=G_{{\mathrm{OSi}}^{x}}\left(1\right)$
m=1	$G\left(2\right)=G_{m}\left(1\right)$	$p_{{\mathrm{OSi}}^{x}}^{c}=0.01,p_{{\mathrm{OSi}}^{x}}^{f}=1.0$	$G\left(11\right)=G_{{\mathrm{OSi}}^{x}}\left(2\right)$
m=10	$G\left(3\right)=G_{m}\left(2\right)$	$p_{{\mathrm{OSi}}^{x}}^{c}=1.0,p_{{\mathrm{OSi}}^{x}}^{f}=0.01$	$G\left(12\right)=G_{{\mathrm{OSi}}^{x}}\left(3\right)$
m=25	$G\left(4\right)=G_{m}\left(3\right)$	$p_{{\mathrm{OSi}}^{x}}^{c}=1.0,p_{{\mathrm{OSi}}^{x}}^{f}=1.0$	$G\left(13\right)=G_{{\mathrm{OSi}}^{x}}\left(4\right)$
λ=0.0	$G\left(5\right)=G_{\lambda }\left(1\right)$	$p_{\mathrm{CR}}^{f}=0.01$	$G\left(14\right)=G_{\mathrm{CR}}\left(1\right)$
λ=0.1	$G\left(6\right)=G_{\lambda }\left(2\right)$	$p_{\mathrm{CR}}^{f}=1.0$	$G\left(15\right)=G_{\mathrm{CR}}\left(2\right)$
λ=0.5	$G\left(7\right)=G_{\lambda }\left(3\right)$	$p_{{\mathrm{Si}}^{x}O^{x}}^{f}=0.01$	$G\left(16\right)=G_{{\mathrm{Si}}^{x}O^{x}}\left(1\right)$
$p_{{\mathrm{CO}}^{x}}^{c}=0.01$	$G\left(8\right)=G_{{\mathrm{CO}}^{x}}\left(1\right)$	$p_{{\mathrm{Si}}^{x}O^{x}}^{f}=1.0$	$G\left(17\right)=G_{{\mathrm{Si}}^{x}O^{x}}\left(2\right)$
$p_{{\mathrm{CO}}^{x}}^{c}=1.0$	$G\left(9\right)=G_{{\mathrm{CO}}^{x}}\left(2\right)$		

## Abbreviations

The following abbreviations are used in this manuscript:

BFM	bond fluctuation model
rBFM	reactive bond fluctuation model
RDF	radial distribution function
nMC, rMC	non-reactive Monte Carlo, reactive Monte Carlo
